# Incontinentia pigmenti burden scale: designing a family burden questionnaire

**DOI:** 10.1186/s13023-019-1234-y

**Published:** 2019-11-26

**Authors:** Charles Taieb, Smail Hadj-Rabia, Jacques Monnet, Mohammed Bennani, Christine Bodemer, the Filière Maladies Rares en Dermatologie

**Affiliations:** 1 French Rare Diseases Healthcare Network Department of Dermatology, Necker Enfants Malades Hospital Paris, APHP, Paris, France; 2 FIMARAD, Hôpital Necker-Enfants Malades, APHP, Paris, France; 3 Department of Dermatology, Reference Center for Genodermatoses (MAGEC), Necker-Enfants Malades Hospital (AP-HP) and Imagine Institute, Paris Descartes-Sorbonne Paris Cité University, Paris, France; 4 French Association of Incontentia Pigmentosa Patients, Paris, France; 5 Qualees, Paris, France

**Keywords:** Incontinentia pigmenti, Disease burden, Quality of life, Orphan disease

## Abstract

**Background:**

Incontentia pigmenti (IP) is a rare multisystem disorder of ectodermal origin comprising skin, dental, ocular and central nervous system features. Symptomatic treatments are adapted to each family according to the patient’s disability. Due to its rarity, the family IP burden in its broadest sense (psychological, social, economic and physical) has not yet been evaluated.

**Aim:**

To design a questionnaire allowing assessing the family burden of IP (F’BoIP).

**Method:**

A questionnaire was developed using a standardized methodology for designing quality of life questionnaires according to the following steps: conception, development, and validation. A multidisciplinary working group was designed, including experts in questionnaire development, dermatologists specialised in IP patient care and representatives of the French IP association. A cultural and linguistic validation into US English was conducted, based on the original French version.

**Results:**

A 20-item conceptual questionnaire was generated. Subsequent confirmatory analyses produced a 20-item questionnaire grouped into four domains, demonstrating internal consistency (Cronbach’s alpha: 0.93), reproducibility and high reliability. The F’BoIP questionnaire significantly correlated with other validated questionnaires: Family Dermatology Life Quality Index (F-DLQI), Perceived Stress Scale (PSS) and SF-12 mental and SF12 physical scores, indicating good external validity.

**Conclusion:**

The F’BoIP questionnaire is the first specific tool to assess the family burden of IP and can be used by both family members of IP patients and by health care professionals. It is a valuable tool which evaluates medical and nonmedical strategies to improve the daily life of families affected by this orphan disease.

## Background

IP, or Bloch-Sulzberger syndrome, is a rare multisystem disorder of ectodermal origin comprising skin, dental, ocular and central nervous system features such as seizures, spastic paralysis, microcephaly and intellectual disability. IP is an X-linked dominant genodermatosis. It affects exclusively female patients and is usually lethal in utero in males [1]. The condition is caused by a genetic mutation in the *IKBKG* gene located on the X chromosome [2]. This gene is known as nuclear factor-kappa B (NF-kappaB) essential modulator (NEMO), required for activating the transcription of factor NF-kappaB. The NF-kappaB pathway plays a central role in the expression of numerous genes pertaining to the immune system, including those involved in embryonic development and the development of bone, skin, mammary glands and the central nervous system (CNS). NF-kappaB activation is defective in IP cells.

Skin manifestations are the first diagnostic signs observed at birth or during early life. They are subdivided into four stages, comprising blisters, hyperkeratotic lesions, hyperpigmentation and, lastly, atrophic lesions [2]. These skin lesions follow Blaschko lines, virtual lines thought to represent the clonal boundaries of cells migrating from the neural crest. While lesions resolve spontaneously, residual hyper- or hypopigmentation can persist throughout life.

Clinical IP diagnosis is based on major and minor criteria associated with the skin lesions [2]. In addition, minor criteria include extracutaneous disease lesions [3, 4]. Its severity is related to ocular or neurological impairment. While no specific treatment is available, symptomatic measures are adapted to each family, according to the patient’s disability. Scientific literature has reported about 1000 cases of IP worldwide [3]..

To date, individual patient disease burden has already been evaluated in many skin diseases, such as psoriasis, infantile haemangioma, hereditary ichthyoses, atopic dermatitis, vitiligo, albinism and palmoplantar keratoderma [5–11].

But, burden and reduced quality of life (QoL) are not only limited to patients, family members and caregivers may also be impacted, and in some situations the QoL of a partner or a parent may even be more impaired than that of the patient. As such, family members and caregivers may experience a major impact on their lives such as physical and mental exhaustion, social disruption, marital problems and financial implications [12, 13].

However, a few instruments to assess the impact of skin diseases on patients’ family members, such as the Family Dermatology Life Quality Index (F-DLQI), have been developed to date [12]. Existing instruments to assess family burden in dermatology have been recently reviewed by Sampogna et al. [14].

According to our literature search, no specific instrument exists to assess the burden and impact on QoL of parents of IP patients. However, such a tool would be useful for both parents of IP patients and clinicians in charge of patient management. The instrument could, to begin with, serve as a way of describing the IP parents’ perceptions. Secondly, it could be used as a tool in the follow-up of any changes in the patient’s medical and non-medical care. As part of its research activities, the French national network of expert centres for rare skin disorders has developed three different questionnaires for rare skin diseases [7, 10, 11].

The objective of the present study was to develop a self-administered questionnaire in order to assess the individual family burden in an IP patient cohort (F’BoIP). The second objective was to ensure that this questionnaire could be used by as many IP families as possible, following cultural and linguistic validation into US English based on the original F’BoIP.

## Material and methods

The self-administered F’BoIP questionnaire was designed using a standard methodology for designing QoL questionnaires [15]. The methodology was based on three distinct phases: conception, development and validation. To ensure the questionnaire’s validity, a multidisciplinary working group was created including experts in the design and development of questionnaires, such as healthcare professionals (physicians and public-health specialists). Moreover, three “expert parents”, active members of the French Association of IP Patients, participated in this working group.

The questionnaire was built in a question and answer format. Response modalities were determined via expert consensus and took the form of a 7-point Likert scale, often used in self-completion questionnaires: “never” (0), “rarely” (1), “sometimes” (2), “often” (3), “very often” (4), “constantly” (5) and “not concerned” (0). To prevent confusion with any changes in perception due to symptoms related to comorbidities, the majority of questions included the wording “IP of your child.”

### Conception

During the conceptual phase, a series of interviews with dermatologists, patient-reported outcome (PRO) experts and IP parents was conducted to comprehensively collect the parents’ perceptions and complaints as an initial wording report. Based on this initial wording report, the working group drew up a list of items that were reformulated as simple questions that would be easily understood. Nine interviews of patient family members (both parents, or only mother or father) were then conducted. Patients were selected in collaboration with the French association of IP patients which only proposed the questionnaire to parents of previously diagnosed IP patients. This ensured broad recruitment and the guarantee of diversity in patients in terms of geographical location, as well as age and sociological status. Thus, a semi-structured questionnaire was built. It discussed specific themes using closed-ended questions with a choice of predetermined answers. The final choice of questions was made by the working group who analysed the initial wording report semantically. The wording of each question was examined, thereby allowing regrouping questions if their similarities were proved too strong.

### Development

During this phase the conceptual questionnaire was administered to a random sample of parents of IP patients (*n* = 114). This was followed by an exploratory factor analysis in order to reveal latent constructs, assigning each item to its respective domain or dimension.

A principal component analysis using a varimax orthogonal rotation was performed, to determine to which domain or dimension each question belonged [16]. Whenever questions could be linked to several dimensions, questions were allocated to the dimensions deemed to be the most semantically relevant by the expert working group.

### Validation

#### Internal validation

To evaluate the questionnaire’s internal consistency, the homogeneity of the items in each dimension was tested using Cronbach’s alpha coefficient [8]. Scores in the higher ranges (>0.7) generally suggested that the items measured the same entity, indicating good homogeneity.

To demonstrate the questionnaire’s unidimensionality, a higher order factor confirmatory analysis was performed, aiming to confirm that dimensions could be combined into one single score. The model’s goodness-of-fit was assessed using several criteria, the Bentler comparative fit index and Bentler-Bonett non-normed fit index [17]. Criteria for a model’s goodness-of-fit were defined as a Bentler comparative fit and Bentler-Bonett non-normed fit index both >0.90. The root mean square error of approximation (RMSEA) was to be about 0.05 or at the very least <0.08, with 0.05 contained within the confidence interval.

#### External validation

To determine the questionnaire’s external validity, all participants were asked to complete three validated self-administered questionnaires: a 12-Item Short Form Health Survey (SF12), a F- DLQI questionnaire and a PSS [12, 18, 19].

The SF12 is a short version of the SF-36, a well-known quality of life tool. Based on 12 questions, a physical composite score (PCS) and mental composite score (MCS) were calculated.

The FDLQI is a questionnaire designed for adults (more than 16 years of age), family members or partners of patients (of any age) with any skin disease. The F-DLQI represents the sum of all scores (0–30); results can equally be expressed as percentages (0–100%).

The PSS, developed by Cohen et al. in 1983, is the most widely-used psychological instrument for measuring the perception of stress. Composed of 10 items, rated from “never” to “often”, the PSS measures the degree to which situations in one’s own life are perceived as stressful. The total score ranges from 10 to 50; the higher the score, the higher the stress.

Pearson correlation was calculated to assess the validity between the F’BoIP and the three other questionnaires.

All data were analysed using SAS software Version 9.4 (SAS Institute, Cary, NC, USA) for Windows, with a significance level set at 0.05.

#### Test-retest validation

To assess reproducibility, test-retest analyses were carried out. Participants answered the questionnaire once and then again after a 10 to 12-day interval. Using these means, answers obtained were compared and the reliability of measurements confirmed.

#### Translation, cross-cultural adaptation and cognitive debriefing

The validated methodology was applied to generate a US English-language version, according to the recommendations of the ISPOR task force [20]. This process, comprising a meticulous 9-step procedure, refined the translation while taking into account subtle nuances of the source document.

The different steps employed are summarized in Table 1.

**Table 1:** Principles of Good Practice for the Translation and Cultural Adaptation Process for Patient-Reported Outcomes (PRO) Measures [20]

Stage	Details
Preparation	Evaluation of the source text from a linguistic and cultural point of view including definition of concepts
Forward translations	Forward translation into the required target language by two independent translators
Reconciliation	Comparison of the two forward translations to provide the best adapted and to produce a draft versions of the text
Back translation	Translation of the draft forward translation back into the targeted language without reference to the original language
Back translation review	Comparison of the original text and the back translation to verify if changes are required to the draft forward version
Analysis and implementation of back translation review report	Analysis of the back translation review report to verify if changes are required to draft forward version
Pilot testing	Clinical review and cognitive debriefing
Review of cognitive debriefing or clinical review results	Review of results from the cognitive debriefing or clinical review to identify translation modifications necessary for improvement

## Results

### Conception

This phase involved IP patients’ parents who expressed and shared their perceptions and complaints regarding their children’s IP. The research resulted in an initial wording report. Several exchanges and one-to-one discussions between IP dermatologists, sociologists and experts in healthcare-related outcomes contributed to further consolidating this initial verbatim report. A 21-item questionnaire was then drawn up, thereby defining the conceptual questionnaire.

### Development

The invitation to participate in this study was addressed to 114 families through the patient association. Of the 114 invited participant parties, 82 participated and activated the questionnaire, 63 agreed to participate after having read the information to participants and 2 did not complete the entire questionnaire. Thus, 61 questionnaires were evaluable for our study (Table 2). The total number of children affected was 71 as each responder could have had one or more children affected (52 responders had one child, 8 had 2 and one responder had 3 children affected).

**Table 2:** Socio-economic status and age of parents, disease manifestations and management details of IP-affected children

Items	N	Population	data missing	%
One of the parents with IP	26	61	0	42.6%
Living in a couple	31	61	0	50.8%
Living alone	20	61	0	32.8%
Average parents’ age		41.7 ± 12.8		
High income group	16	61	0	26.2%
Low income group	8	61	0	13.1%
Unemployed	17	61	0	27.9%
Higher education qualification	18	61	0	29.5%
No qualifications	5	61	0	8.2%
100% health insurance cover	7	53	8	13.2%
Diagnosis made by a dermatologist	49	53	8	80.3%
Clinical signs				
ocular	52	61	9	85.3%
dental	50	61	11	82.0%
neurological	20	61	41	32.8%
cutaneous	18	61	43	29.5%
Satisfied overall with care provided	27	57	4	47.4%
Satisfied with medical care provided by your physician	25	50	11	50.0%
Followed by an ophthalmologist	52	61	0	85.3%
Followed by a psychologist	12	61	0	19.7%
Followed by a speech therapist	17	61	0	27.9%
Followed by an occupational therapist	11	53	0	20.8%
Followed by a massage therapist	15	61	0	24.6%
Attended a therapy education programme	7	61	0	11,5%
Contact with an association	45	61	0	73.8%

In 26 of all cases, the mother was affected and in 7 cases a brother or a sister. The different scores were calculated for both groups: the 26 families where one of the parents was affected by IP compared to the 35 families with no affected parents. However, the sample was too small to show any significant difference. QoL was slightly more impacted if one of the parents was affected; in this group also stress, evaluated by the PSS questionnaire, appeared somewhat more pronounced.

Conversely, burden was less important if one of the parents was affected. This may be due to the fact that the affected parent anticipated potential difficulties resulting in a less importantly perceived burden (Table 3).

**Table 3:** Comparison of mean scores of newly developed F’BoIP questionnaire compared to F-DLQI, stress evaluated by PSS, and SF12 Mental and Physical dimension

	New score	FDLQI	STRESS	SF12 Mental dimension	SF12 Physical dimension
GLOBAL	32.31 ± 19.28	6.62 ± 8.51	28.48 ± 8.34	50.64 ± 9.52	39.90 ± 11.83
*N* = 61					
Parents not affected	33.83 ± 18.66	6.35 ± 8.57	27.00 ± 9.07	49.61 ± 9.39	42.04 ± 12.66
*N* = 35					
Parents affected	30.27 ± 20.26	7.00 ± 8.59	30.58 ± 6.81	52.10 ± 9.71	36.86 ± 10.04
*N* = 26					
*p*-value	0.2401	0.3890	0.0537	0.1654	0.0504

### Exploratory factor analysis

The exploratory analysis was performed on 21 items in order to test the questionnaire’s robustness. According to standardized regression analysis, each group of questions was assigned a dimension, with four dimensions highlighted as follows (Table 4):

- Dimension 1, with eight questions regarding social life and family life
- Dimension 2, with six questions regarding professional life and renunciation
- Dimension 3, with five questions regarding daily life
- Dimension 4, with two questions regarding economic impact

**Table 4:**
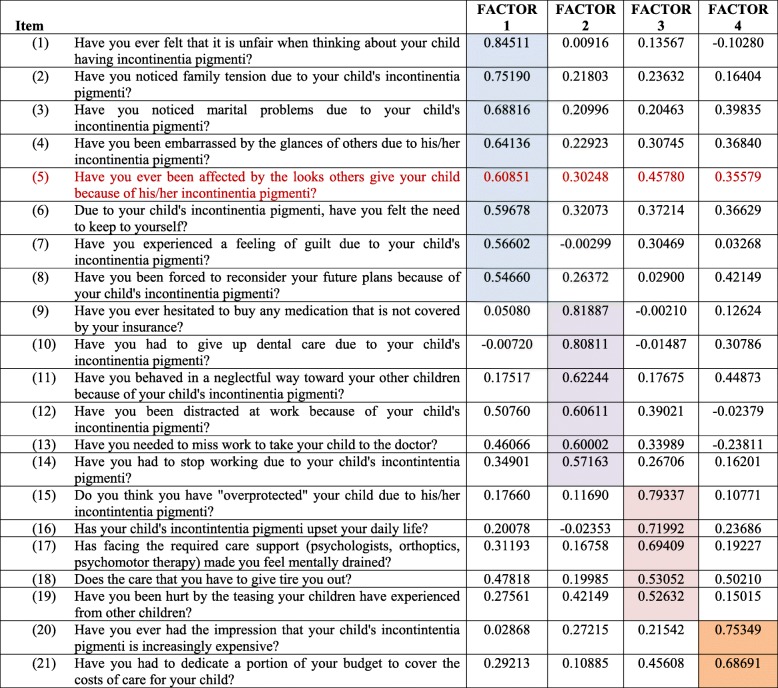
Loading of questions on factors after rotation

Loadings (correlation coefficients between questions and factors) were computed to allow facilitating the interpretability of factors. A loading of more 0.5 indicated that the couple question-factor was strongly related to each other. A question that did not have a loading of more 0.5 was not particularly related to any of the selected factors

Questions were reordered to show those corresponding to Factor 1 (blue), to Factor 2 (yellow), to Factor 3 (pink), and to Factor 4 (green). Factors were interpreted by looking at the common theme among questions that belong to the same factor

The confirmatory analysis showed that the question *“Have you ever been affected by the looks others give your child because of his/her IP?”* was non-relevant. This question was therefore removed, resulting in a final questionnaire of 20 items, grouped into four dimensions. Thus, only Dimension 1 was modified from eight to seven questions.

### Validation

#### Internal validation

The questionnaire’s unidimensionality was confirmed by a higher order factor analysis, as shown in Table 4. The practical goodness-of-fit indices were acceptable, with a Bentler comparative fit index of 0.9598 and Bentler-Bonett non-normed fit index of 0.9526 (Table 5). Based on these indicators, the model was proven to be well-adjusted and well-fitted; the four dimensions could be grouped together into one single overall score, the F’BoIP.

**Table 5:** Model assessment parameters

	Synthesis of adjustments made		
	Higher order factor criteria	Required	Obtained
Absolute index	Ratio of chi-sq to degrees of freedom	< 5	1.17
Absolute index	Pr > Khi-2	Not significant	0.0701
Absolute index	Normalized RMR	< 0.05	0.0778
Absolute index	Goodness of fit index (GFI)	> 0.8	0.805
Parsimonious index	Adjusted goodness of fit index (AGFI)	> 0.8	0.7457
Parsimonious index	RMSEA estimate	About 0.05 and at the very least under 0.08.	0.0531
Parsimonious index	Inferior limit of the 90% RMSEA confidence interval (CI)		0
Parsimonious index	Superior limit of the 90% RMSEA confidence interval (CI)		0.0824
Parsimonious index	Akaike information criterion	The lowest value possible of the models tested	286.2104
Incremental index	Bentler comparative fit index	> 0.9	0.9598
Incremental index	Bentler-Bonett non-normed index	> 0.9	0.9526

RMSEA: root mean square error of approximation

Model-fit tests were applied to assess the model’s validity. Multiple models were tested, and those with lowest RMSEA and BIC were selected

Cronbach’s alpha coefficient reached 0.93 for the entire questionnaire, reflecting its excellent internal coherence. Moreover, intra-dimensional coherences showed good reliability (α >  0.72).

#### External validation

The F’BoIP questionnaire highly correlated with the validated F-DLQI and PSS questionnaires, as well as the SF12 mental and SF12 physical scores (Table 6). Correlation coefficients between F’BoIP and the validated questionnaires were relatively high, confirming its external validity. F’BoIP’s correlation with the SF12 physical score was weaker than that obtained with the SF12 mental score.

**Table 6:** Correlations between scores

Pearson correlation coefficients, *N* = 58^a^ Probability > |r| under H0: Rho = 0					
	DLQI	PSS	SF12 mental score	SF12 physical score	F′ BoIP
F-DLQI	1	0.61318 (<.0001)	−0.58469(<.0001)	−0.59583(<.0001)	0.69608(<.0001)
PSS	0.61318 (<.0001)	1	−0.84159 (<.0001)	−0.38021 (0.0032)	0.63063 (<.0001)
SF12 mental score	−0.58469 (<.0001)	−0.84159 (<.0001)	1	0.23431 (0.0767)	−0.57441 (<.0001)
SF12 physical score	−0.59583 (<.0001)	−0.38021 (0.0032)	0.23431	1	−0.47374 (0.0002)
F′ BoIP	0.69608 (<.0001)	0.63063 (<.0001)	−0.57441 (<.0001)	−0.47374 (0.0002)	1

Correlation coefficients between F’BoIP and 4 four self-administrated questionnaires are shown

Significant *p*-values indicate that scores are strongly related to previously validated scores, confirming the external score validity

^a^Correlation was assessed using data from 58 subjects (instead of 61), because of missing data for F-DLQI, SF12 and STRESS for three subjects

Moreover, the study confirmed the following correlations shown for previously developed questionnaires:

- A closer correlation was expected and reached with the F-DLQI questionnaire and the PSS questionnaire (specific instruments) than with SF12 (generic instrument). Furthermore, a correlation with the F-DLQI, specific to dermatology, proved to be the closest.
- A greater correlation was expected and shown with the mental dimension of SF12 than with the physical dimension. Indeed, the physical dimension has been affected to a limited extent as most of the families were quite young. Thus, QoL impairment concerned more a psychological fatigue rather than a physical fatigue.

Moreover, results obtained were in line with the COSMIN classification of correlations with external measures [8, 9].

#### Test-retest analysis

The test-retest reliability was assessed, based on test results from 20 subjects undergoing testing on both Day 0 and Day 10. Results demonstrated a very good reproducibility, with an intra-class correlation of each dimension exceeding 0.85 for each domain***.***

#### Cognitive debriefing, translation and cross-cultural adaptation

Cognitive debriefing did not result in any changes regarding the questions’ wording. The original French version of the F’BoIP questionnaire was translated and underwent linguistic and cultural validation into US English.

#### F’BoIP scoring

The total F’BoIP score was obtained by summing up scores for each of the 20 questions as defined in the aforementioned method description, with “never” or “not applicable”, which scored 0, “rarely” 1, “sometimes” 2, “often” 3, “very often” 4, and “constantly” 5.

A final, validated version of the questionnaire is given in Table 7.

**Table 7:** Final validated version of the IP Family Burden questionnaire

		Score from 0 to 5
1	Have you ever felt that it is unfair when thinking about your child having incontinentia pigmenti?	
2	Have you noticed family tension due to your child’s incontinentia pigmenti?	
3	Have you noticed marital problems due to your child’s incontinentia pigmenti?	
4	Have you been embarrassed by the glances of others due to his/her incontinentia pigmenti?	
5	Due to your child’s incontinentia pigmenti, have you felt the need to keep to yourself?	
6	Have you experienced a feeling of guilt due to your child’s incontinentia pigmenti?	
7	Have you been forced to reconsider your future plans because of your child’s incontinentia pigmenti?	
8	Have you ever hesitated to buy any medication that is not covered by your insurance?	
9	Have you had to give up dental care due to your child’s incontinentia pigmenti?	
10	Have you behaved in a neglectful way toward your other children because of your child’s incontinentia pigmenti?	
11	Have you been distracted at work because of your child’s incontinentia pigmenti?	
12	Have you needed to miss work to take your child to the doctor?	
13	Have you had to stop working due to your child’s incontintentia pigmenti?	
14	Do you think you have “overprotected” your child due to his/her incontintentia pigmenti?	
15	Has your child’s incontintentia pigmenti upset your daily life?	
16	Has facing the required care support (psychologists, orthoptics, psychomotor therapy) made you feel mentally drained?	
17	Does the care that you have to give tire you out?	
18	Have you been hurt by the teasing your children have experienced from other children?	
19	Have you ever had the impression that your child’s incontintentia pigmenti is increasingly expensive?	
20	Have you had to dedicate a portion of your budget to cover the costs of care for your child?	

With: 0 = never/not applicable, 1 = rarely, 2 = sometimes, 3 = often, 4 = very often, 5 = constantly

## Discussion

Skin diseases do not only psychosocially affect the patient, but also impact the entire family’s functioning. Today, the individual disease burden is increasingly investigated. “Individual burden” accounts for the broadest aspects of disease-related disability, covering psychological, physical, social and economic factors, simultaneously taking into account QoL, community integration, organization of everyday life, as well as medical resource consumption. Using questionnaires may allow evaluating this overarching burden, especially among patients with a particular disease [5–11].

IP impacts the family’s lifestyle and quality of life heavily. As a result, family members have to reorganize their life, overcome the stress of diagnostic procedures and integrate the notion of chronicity and absence of curative treatments into their daily life.

Based on advances made in QoL research over the last decades, health-care professionals and regulatory agencies, such as the US Food and Drug Administration (FDA) and the European Medicine Agency (EMA), currently face complex issues related to the development of health-related quality of life claims for both product labelling and promotion [6]. In this context, Leidy et al. generated recommendations for the healthcare industry to ensure that all health-related QoL claims are based on rigorously-designed studies, with appropriate methodology and instruments [19]. Development in the clinical research field has led to the widespread use of questionnaires and this trend is most likely to continue in the near future. The reason for this is the increasing relevance of data that are both closer to clinical practice and increasingly needed to achieve market access. At present, QoL, patient wellbeing, and patient-centred outcomes are requested by reimbursement agencies, such as NICE in the UK and IQWIG in Germany [9].

To our knowledge, no specific instrument exists that is able to assess the overall burden of parents of children with IP. This paper provides an easy-to-use questionnaire which assesses the individual disease burden of parents of IP patients. The questionnaire is currently available in French and US English and can be translated and culturally and linguistically validated into other languages, such as German, Spanish, etc. The 9-step methodology required to generate linguistically-validated and cross-culturally-adapted F’BoIP versions into other languages is well-established. With its 20 items and six possible answers for each question, our questionnaire is relatively short, understandable and easy to use. F’BoIP is a robust tool with an internal consistency exceeding the minimum reliability criterion of 0.90 for individual analysis.

The main limitation of our study is the relatively small sample size of 61 parents. However, as IP is a rare disease, a small sample size is not surprising.

We believe that the objective evaluation of the IP burden will enhance communication between patients, parents and healthcare providers, thereby improving information transfer and creating a real opportunity for practitioners to gain better understanding of certain issues brought up by the patients or their families.

Moreover, F’BoIP may allow the development of new patient management approaches and the improvement of existing patients’ health, care and daily-life.

## Conclusions

F’BoIP demonstrates feasibility, validity and discriminant reliability. It can therefore be used to understand the multidimensional nature of IP better, in addition to the individual burden of the parents of patients suffering from this condition. Moreover, it may play a role in the decision-making process. Additional research to develop a version of the instrument for children to use in the future is currently ongoing.

## Data Availability

The datasets used and/or analyzed during the current study are available from the corresponding author on reasonable request.

## Abbreviations

BIC: Bayesian information criterion
CNS: Central nervous system
EMA: European Medicine Agency
FDA: Food and Drug Administration
F-DLQI: Family-Dermatology Life Quality Index
IP: Incontinentia pigmenti
IQWIG: Institut für Qualität und Wirtschaftlichkeit im Gesundheitswesen
MCS: Mental composite score
NICE: National Institute for Health and Care Excellence
PCS: Physical composite score
PRO: Patient-reported outcome
PSS: Perceived Stress Scale
QoL: Quality of Life
RMSEA: Root mean square error of approximation
US: United States
